# Strong constraint on modelled global carbon uptake using solar-induced chlorophyll fluorescence data

**DOI:** 10.1038/s41598-018-20024-w

**Published:** 2018-01-31

**Authors:** Natasha MacBean, Fabienne Maignan, Cédric Bacour, Philip Lewis, Philippe Peylin, Luis Guanter, Philipp Köhler, Jose Gómez-Dans, Mathias Disney

**Affiliations:** 1 Laboratoire des Sciences du Climat et de l’Environnement, LSCE/IPSL, CEA-CNRS-UVSQ, Université Paris-Saclay, F-91191 Gif-sur-Yvette, France; 2 Present Address: University of Arizona, School of Natural Resources and the Environment, Tucson, Arizona 85719 USA; 3 NOVELTIS, Labège, France; 4 Department of Geography, University College London, Pearson Building, Gower Street, London, WC1E 6BT London, UK; 5 NERC National Centre for Earth Observation (NCEO), Swindon, UK; 6 Helmholtz Centre Potsdam-GFZ German Research Centre for Geosciences, Potsdam, Germany; 7 California Institute of Technology (Caltech), Division of Geological and Planetary Sciences, Pasadena, CA USA

## Abstract

Accurate terrestrial biosphere model (TBM) simulations of gross carbon uptake (gross primary productivity – GPP) are essential for reliable future terrestrial carbon sink projections. However, uncertainties in TBM GPP estimates remain. Newly-available satellite-derived sun-induced chlorophyll fluorescence (SIF) data offer a promising direction for addressing this issue by constraining regional-to-global scale modelled GPP. Here, we use monthly 0.5° GOME-2 SIF data from 2007 to 2011 to optimise GPP parameters of the ORCHIDEE TBM. The optimisation reduces GPP magnitude across all vegetation types except C4 plants. Global mean annual GPP therefore decreases from 194 ± 57 PgCyr^−1^ to 166 ± 10 PgCyr^−1^, bringing the model more in line with an up-scaled flux tower estimate of 133 PgCyr^−1^. Strongest reductions in GPP are seen in boreal forests: the result is a shift in global GPP distribution, with a ~50% increase in the tropical to boreal productivity ratio. The optimisation resulted in a greater reduction in GPP than similar ORCHIDEE parameter optimisation studies using satellite-derived NDVI from MODIS and eddy covariance measurements of net CO_2_ fluxes from the FLUXNET network. Our study shows that SIF data will be instrumental in constraining TBM GPP estimates, with a consequent improvement in global carbon cycle projections.

## Introduction

The terrestrial carbon, C, sink remains the most uncertain component of the annual global carbon budget^1^. Uncertainty in its strength and location contributes to high terrestrial biosphere model (TBM) spread in future C sink projections between models^2^. Accurate net CO_2_ flux projections rely on model ability to determine gross C fluxes. However, TBM inter-comparisons have shown strong discrepancies in gross C uptake (or gross primary production – GPP) related to both variability in growing season length and peak season magnitude^3,4^. One source of uncertainty in TBM simulations is due to fixed (often uncertain) parameter values. Significant progress has been made in using carbon cycle-related observations to constrain TBM parametric uncertainty via Bayesian data assimilation (DA) methods^5–10^. These datasets have included eddy covariance measurements of net ecosystem exchange (NEE)^11^, satellite-derived measures of vegetation dynamics^12–14^, and ground-based atmospheric CO_2_ concentration data. However, while there is considerable improvement in the simulation of leaf phenology and/or NEE in these studies, there is often a remaining model-data discrepancy in the gross C fluxes.

Satellite-derived measures of sun-induced chlorophyll fluorescence (SIF) offer a promising new direction to constrain simulated GPP at multiple scales^15^. SIF is strongly linked to GPP via its association with chlorophyll *a* absorption in plant photosynthetic machinery. The SIF-GPP relationship has been assessed at multiple scales using both process-based modelling and *in situ* and satellite observations. Frankenberg C. *et al*.^16^ and Guanter L. *et al*.^17^ were the first to report a linear relationship at global scale between monthly satellite-derived SIF data from the Greenhouse Gases Observing Satellite (GOSAT) and an up-scaled FLUXNET GPP product^18^. Similarly, GOME-2 SIF data (Global Ozone Monitoring Experiment-2 onboard the MetOp-A satellite) have also been shown to be linearly correlated with eddy covariance flux tower GPP measurements of gross C fluxes^19–21^. The consistency of this proposed linear SIF-GPP relationship across multiple spatial and temporal scales has been debated in the community. Several site-level studies found that while the SIF-GPP relationship in instantaneous leaf level measurements is non-linear and dependent on vegetation type, it becomes linear upon aggregation to the canopy, and at daily to seasonal time-scales^21–25^. Linear relationships have been even been found between flux tower GPP and instantaneous values derived from the OCO-2 (Orbiting Carbon Observatory) SIF product across a range of sites^15,26,27^. Most of these studies suggested that although temporal and/or spatial aggregation appears to result in increasing linearity in the SIF-GPP relationship, the slope remains biome-specific due to differences in canopy structure and biochemistry^17,23,25–27^ (though see ref.^15^).

Meanwhile, several modelling groups have used SIF data to optimise fluorescence model parameters^23^ and simple carbon cycle model physiology and leaf growth parameters^28^; to evaluate TBM GPP^29,30^; and to constrain TBM GPP outputs^31^. However, efforts are still underway to implement a mechanistic fluorescence models in TBMs^32^. Here, we explore a simpler approach to using SIF data to reduce TBM uncertainty by assuming a biome-specific linear relationship between SIF and GPP at broad spatial and temporal scales. More specifically, we use monthly aggregated 0.5 × 0.5° GOME-2 SIF data to optimise PFT-dependent GPP-related parameters in the ORCHIDEE TBM. Our key objective was to investigate if SIF data, and the simple linear SIF-GPP relationship, can be used to constrain regional to global, and monthly to annual modeled GPP. If successful, this simple approach – compared to the implementation of fluorescence processes in models – would allow a new, easy-to-implement method for using SIF data to optimise TBMs.

## Results

### Impact of the optimisation on GPP spatio-temporal patterns

**Figure 1:**
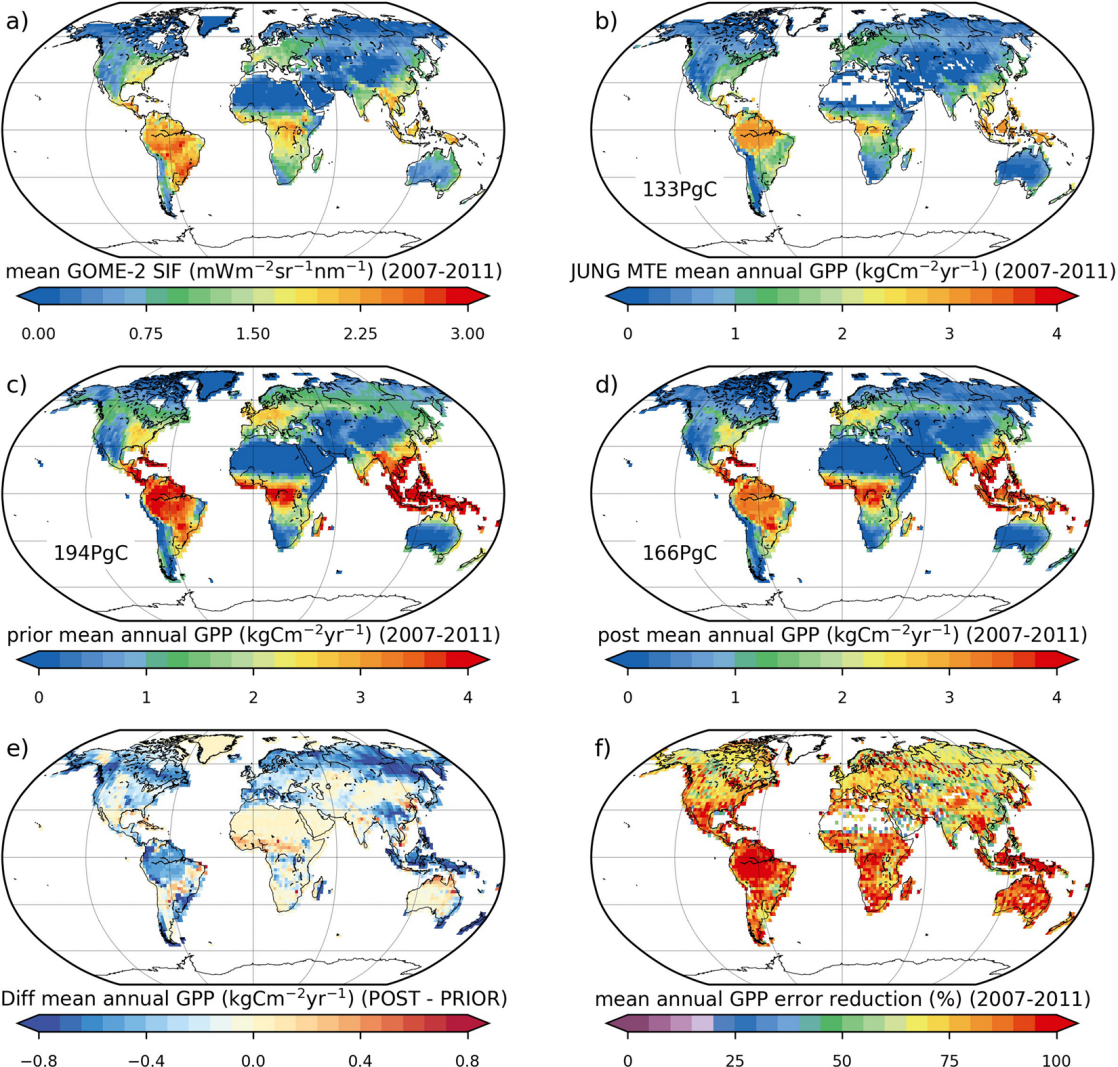
Global mean annual sum (2007–2011) and spatial distribution of: (**a**) GOME-2 SIF; (**b**) JUNG up-scaled FLUXNET data-driven GPP product^18^; (**c**) ORCHIDEE prior GPP; (**d**) ORCHIDEE posterior GPP; (**e**) difference in ORCHIDEE simulated GPP (posterior – prior); (**f**) reduction in GPP uncertainty (1*σ*). The maps were created from the ORCHIDEE model simulations performed in this study, GOME-2 SIF data, and the JUNG product, using the Python programming language v2.7.13 (Python So ware Foundation – available at http://www.python.org) Matplotlib (v2.0.2) plotting library54 with the Basemap Toolkit (http://matplotlib.org/basemap/). See Section on Data Availability for GOME-2 SIF and JUNG product availability, the ORCHIDEE model licence information and ORCHIDEE code availability.

Optimising the ORCHIDEE parameters related to photosynthesis, phenology and the linear SIF-GPP relationship using GOME-2 SIF data resulted in a considerable reduction in GPP at global scale (Fig. 1c,d, and e and Table 1 row 2). Specifically, strong reductions occurred in both densely forested regions of the boreal high northern latitudes, and tropical regions in South America and Southeast Asia (Fig. 1e and Table 1). There was a 28.8 PgCyr^−1^ reduction in the global mean (2007–2011) annual total GPP (Fig. 1c,d and Table 1); therefore, the posterior ORCHIDEE estimate of 165.6 PgCyr^−1^ is more in line with the JUNG estimate of 133.4 PgCyr^−1^ (Fig. 1b) compared to the prior value of 194.4 PgCyr^−1^. At biome level, the mean annual total GPP decreased from 88.6 to 67.1 PgCyr^−1^ in the temperate and boreal ecosystems, from 92.2 to 86.1 PgCyr^−1^ in the tropics, and from 13.6 to 12.4 PgCyr^−1^ in arid biomes (Table 1). In the temperate and boreal biomes, the larger share of the reduction in GPP occurred in boreal zones (classes D and E in the KG classification – see Methods): Temperate region decrease in GPP was only 40% that achieved in boreal zones. The largest decrease in annual GPP per unit area was seen in northern extra tropical latitudes (temperate and boreal biomes) followed by the tropical biome (Fig. 1e and Supplementary Table S2 column 2). This was associated with strong reduction in GPP (>0.5 kgCm^−2^ yr^−1^) for all boreal PFTs as well as temperate and tropical broadleaved evergreen trees (TeBE and TrBE – Supplementary Table S2 and see Fig. 5 for PFT acronym descriptions). For these PFTs, the mean reduction per PFT corresponds to ~40% of the prior temperate forest mean annual budget, ~60–110% of the prior boreal mean annual budget, and ~16% of the prior tropical forest mean. Overall, the optimisation resulted in a ~32% increase in the ratio of productivity per unit area between the tropical and extra-tropical (temperate + boreal) KG biomes (Supplementary Table S2). When considering total PgC, the increase in the productivity ratio was 24% (Table 1). This value increased to ~50% in the ratio per unit area between tropical and boreal-only KG biomes (~40% in total PgC). The posterior ratios (1.28 in total PgC for tropical: temperate + boreal biomes; 2.75 for tropical:boreal) better match the same ratios derived from JUNG dataset (1.22 for tropical: temperate + boreal biomes; and 2.54 for tropical:boreal). The shift in global productivity from high latitudes towards the tropics is broadly consistent with Parazoo N. C. *et al*.^31^, who used GOSAT SIF data to constrain the mean GPP of the TRENDY model inter-comparison outputs.

**Table 1:** Annual GPP optimisation performance metrics – mean across different regions and PFTs (with a grid cell fraction greater than a given fraction – see Methods): (i) Columns 2 and 3: prior and posterior mean annual GPP (2007–2011) in PgC; (ii) Column 4: % reduction in the annual GPP uncertainty (1σ) (2007–2011); (iii) Columns 5 and 6: prior and posterior mean monthly correlation between GPP and SIF (2007–2011) at global scale and for each biome. Biomes are based on the Köppen-Geiger (KG) classification derived by Peel M. C. *et al*.^53^.

Region/PFT	Prior mean annual GPP (PgC)	Posterior mean annual GPP (PgC)	Reduction in annual GPP uncertainty (%)	Prior mean monthly SIF-GPP correlation	Posterior mean monthly SIF-GPP correlation
Global	194.4	165.6	82.8	0.72	0.74
Temperate + boreal KG biome	88.6	67.1	67	0.77	0.77
Tropical KG biome	92.2	86.1	93.4	0.5	0.5
Arid KG biome	13.6	12.4	88.9	0.59	0.61

Interestingly, although arid biome mean annual GPP decreased slightly overall (Table 1), mean annual GPP increased in many dry/semi-arid zones including the Sahel, the dry tropics of North and South America, India, China and northern Australia (Fig. 1), due to a simulated higher GPP for C4 grasses and crops (and despite a decrease in tropical broadleaved deciduous tree productivity – Supplementary Table S2). Again, this result is broadly consistent with Fig. 7a in Parazoo N. C. *et al*.^31^. The increase in C4 GPP partially offset the observed reduction in GPP in tropical rainforests.

The mean uncertainty (1σ) in simulated global annual GPP for the 2007–2011 period was reduced by ~83% (Fig. 1f and Table 1) from 57.2 to 9.8 PgCyr^−1^. This global mean value is consistent with Norton A. J. *et al*.^28^ who achieved a 79% reduction in uncertainty when optimising the leaf growth and physiology parameters of a carbon cycle model. As in Norton A. J. *et al*.^28^, the highest reduction in uncertainty was seen in tropical biomes (~93%), whereas the lowest was found in northern temperate and boreal biomes (~67%) (Fig. 1f and Table 1). Posterior mean uncertainty is 2 PgCyr^−1^ for tropical biomes, 7.3 PgC^−1^ for temperate and boreal biomes, and 0.5 PgCyr^−1^ for arid biomes. The high percentage reduction in uncertainty in all biomes is likely an overestimate. This overestimate is partly caused by the fact we did not account for temporal error correlations; in reality therefore, the information content of the observations is likely lower. Another possible explanation is an overestimate of the prior error. The high prior uncertainty (and therefore strong reduction in uncertainty) in tropical regions is likely related to conservative priors (high uncertainty) for many of the TrBE photosynthesis parameters (PFT 2–1^st^ column in Fig. 4). The considerable amount of noise in the GPP uncertainty reduction (Fig. 1f) is likely due to spatial heterogeneity in sub grid-cell PFT fraction.

**Figure 2:**
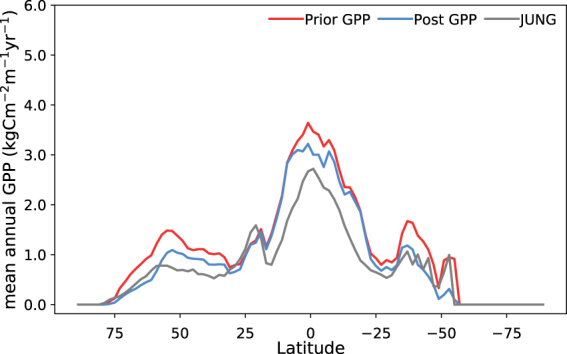
Latitudinal plot of mean annual GPP (kgCm^−2^ yr^−1^) over the 2007–2011 period. The prior simulation is shown in the red curve, the posterior in the blue curve, and the JUNG product in grey.

At global-scale, the continental scale spatial patterns of GPP simulated by the ORCHIDEE model match both the SIF data and the independent JUNG dataset (Figs 1 and 2). However, we can determine if the optimisation had an impact on the finer-scale spatial distribution of simulated GPP by examining the spatial gradients between the prior and posterior in different regions (Figs 1 and 2). The posterior GPP corresponds more closely to JUNG in three regions: the northern extratropics (between ~30–75°N); in the tropics between ~10°N and 10°S; and in the southern hemisphere between 30 and 50°S (Fig. 2) – the same regions which show a strong reduction in GPP. In particular, both the north–south and east–west spatial gradient in the posterior GPP simulations across northern Eurasia appear to better approximate the SIF and JUNG products (Fig. 1). Due to the reduction in GPP in the Amazon, the posterior spatial gradient between the rainforest and Cerrado ecozones in South America is more consistent with SIF data (Fig. 1). In JUNG, there is a more distinct drop in mean annual GPP between the Amazon rainforest and Cerrado region to the southwest (Fig. 1). However, discrepancies between the model and both the JUNG and SIF products remain in the Caatinga, Cerrado and semi-deciduous forests of SE Brazil and in the north-south gradients in sub-Saharan Africa. Greater constraint on the regional to continental scale GPP spatial distributions may be hindered by the underlying PFT maps and the fact we optimised all parameters per PFT, rather than optimising all the parameters for each PFT in each grid cell for all cells. However, this remains both computationally unfeasible for the moment, and conceptually difficult given most TBMs are structured around the PFT-level parameterizations.

**Figure 3:**
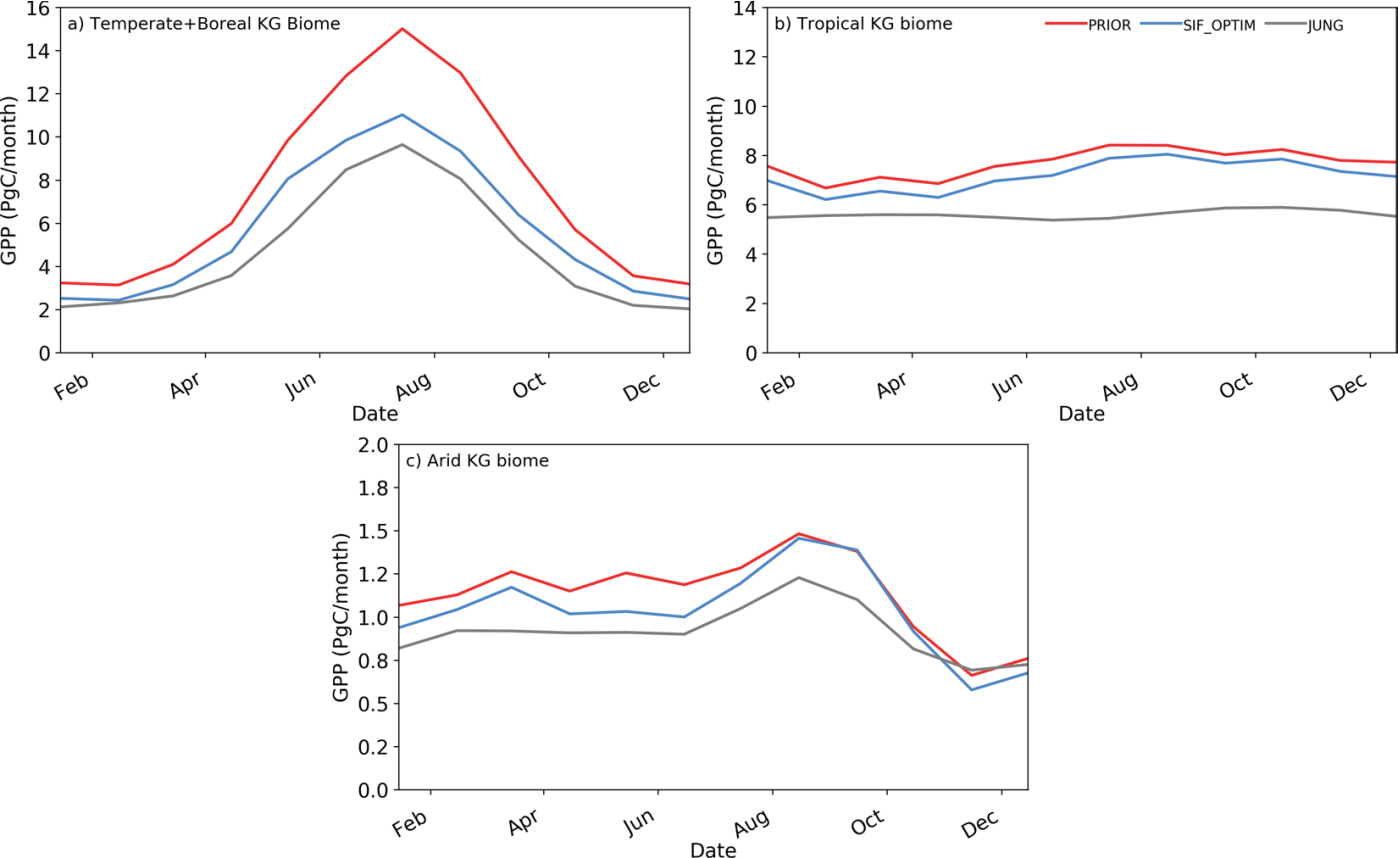
Mean monthly GPP seasonal cycle over 2007–2011 period (PgC/month) for: (**a**) temperate and boreal Köppen-Geiger (KG) biomes (approximately equivalent to northern hemisphere >60°N); (**b**) tropical KG biomes (approximately equivalent to tropical latitudes 30°S to 30°N); (**c**) arid KG biomes. The prior simulation is shown in the red curve, and the posterior in blue. The grey curve shows a comparison with the JUNG up-scaled FLUXNET data-driven GPP product by Jung M. *et al*.^18^. Köppen-Geiger classification based on Peel M. C. *et al*.^53^.

The mean monthly correlation between modelled GPP and GOME-2 SIF did not increase considerably as a result of the optimisation. The optimisation only resulted in a slight adjustment to the timing of seasonal cycle for each biome – resulting in a slightly shorter growing season length in the extratropics (Fig. 3) – and no change in phase. There was little change in the global grid cell mean correlation, nor the average across tropical, arid and temperate/boreal Köppen Geiger biomes (Table 1 columns 4 and 5), nor the average correlation for each PFT (Supplementary Table S3). The lack of improvement in the correlations is explained by the fact the prior correlations were already reasonably high. At PFT level, increases in R value that exceeded 0.04 were found for both boreal broadleaved deciduous trees (BoBD), and C3 grasses and crops (Supplementary Table S3). The decrease in mean annual GPP appears to be mostly associated with a decrease in peak growing season GPP in the northern latitudes (Fig. 3a). A smaller decrease in GPP magnitude is observed across the year for tropical and arid biomes (Fig. 3b,c). The seasonality in tropical regions does not match that of the data-driven JUNG estimate; however, this could not have been corrected by the optimisation due to the lack of an evergreen phenology module or time-varying physiological parameters in the current version of ORCHIDEE. Finally, the optimisation did not result in any discernible change in the long-term (1990–2012) global trend of increasing C uptake, which is ~0.64 PgCyr^−1^. The optimisation also did not change the global-scale or biome level long-term inter-annual variability (IAV) magnitude (3% reduction in global IAV standard deviation) or phase (global-scale correlation with JUNG decreased from 0.44 to 0.4).

### SIF optimisation constraint on the parameter values and uncertainty

**Figure 4:**
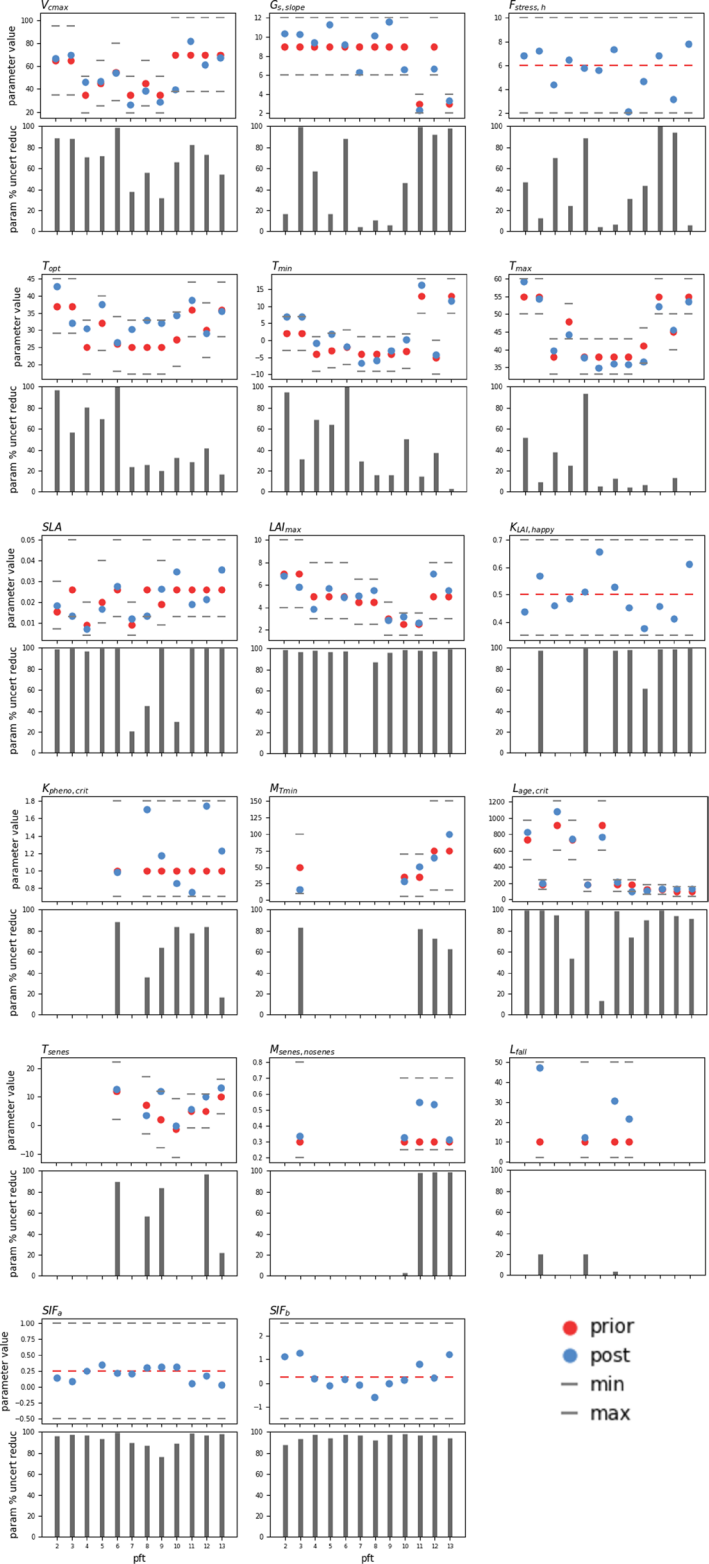
Summary of prior and posterior parameter values and the associated reduction in uncertainty for each PFT and each parameter. Grey dashed lines denote the maximum and minimum bounds, red circles the prior value (dashed line if uniform across all PFTs), blue circles the posterior value and grey bars the reduction in uncertainty. PFT number labels are as follows: 2: TrBE – tropical broadleaved evergreen; 3: TrBR – tropical broadleaved raingreen; 4: TeNE – temperate needleleaved evergreen; 5: TeBE – temperate broadleaved evergreen; 6: TeBD – temperate broadleaved deciduous; 7: BoNE – boreal needleleaved evergreen; 8: BoBD – boreal broadleaved deciduous; 9: BoND – boreal needleleaved deciduous; 10: NC3 – natural C3 grass; 11: NC4 – natural C4 grass; 12: AC3 – C3 crops; 13: AC4 – C4 crops.

The optimisation resulted in greater than 50% reduction in uncertainty for 111 out of a total of 172 parameters (Fig. 4). Despite the fact that the phase of the mean GPP seasonal cycle has not changed considerably between the prior and posterior simulations, the growing season length is slightly shorter post-optimisation in the northern extratropics (Fig. 3), and most phenology parameters were well constrained by the optimisation. Aside from *V*_*cmax*_, the phenology parameters were generally better constrained compared to the photosynthesis parameters. A >50% reduction was achieved for both *V*_*cmax*_ and for all the phenology parameters, except *L*_*fall*_, for the majority of PFTs optimised (Fig. 4; see Table 2 for a description of the parameters). Our results are contrary to Verma M. *et al*.^26^ and Koffi E. *et al*.^32^ who both found limited sensitivity of *V*_*cmax*_ to SIF using the SCOPE (Soil Canopy Observation, Photochemistry and Energy fluxes) model^33,34^. The slope and intercept of the SIF–GPP linear relationship (*SIF*_*a*_ and *SIF*_*b*_ parameters) were also highly constrained (>70% reduction in uncertainty) across all PFTs. Despite the high reduction in phenology parameter uncertainty, the timing (phase) of the GPP seasonal cycle was not altered dramatically by the optimisations. This was most likely due to the fact that the ORCHIDEE model already captures leaf seasonality well (Fig. 3); therefore, this suggests there was limited room for improvement in the posterior mean phenology values. However, it is also likely that phenology is insensitive to monthly SIF data given leaf onset and senescence occurs over a period of days rather than weeks. As found in previous studies using MODIS NDVI to optimise ORCHIDEE phenology-related parameters^14^, the optimisation resulted in an earlier start of leaf senescence, as evidenced by the higher values of *T*_*senes*_ and *M*_*senes,nosenes*_ across most PFTs.

**Table 2:** Description of the parameters used in the optimisation and their PFT dependence (see Fig. 4 for a description of PFT number labels and descriptions).

Parameter	Description	PFT
***Photosynthesis***		
*V*_*cmax*_	Maximum carboxylation rate (µmol·m^−2^·s^−1^)	All
*G*_*s,slope*_	Ball-Berry slope	All
*T*_*opt*_	Optimal photosynthesis temperature (°C)	All
*T*_*min*_	Minimum photosynthesis temperature (°C)	All
*T*_*max*_	Maximum photosynthesis temperature (°C)	All
*F*_*stress,h*_	Parameter reducing the hydric limitation of photosynthesis	All
***Phenology***		
*SLA*	Specific leaf area (m^2^·g^−1^)	All
*LAI*_*max*_	Maximum LAI	All
*K*_*LAI,happy*_	LAI threshold to stop using carbohydrate reserves during growth	All
*K*_*pheno,crit*_	Multiplicative parameter of the threshold that determines the start of the growing season	6, 8–13
*M*_*Tmin*_	Time since moisture minimum for leaf growth	3, 10–13
*L*_*age,crit*_	Average critical age of leaves (days)	All
*T*_*senes*_	Temperature threshold for senescence (°C)	6, 8–13
*M*_*senes,nosenes*_	Moisture threshold for senescence	3, 10–13
*L*_*fall*_	Rate of leaf fall during senescence	3, 6, 8, 9
***GPP – SIF relationship***		
*SIF*_*a*_	Slope parameter in the linear GPP–SIF relationship	All
*SIF*_*b*_	Intercept parameter in the linear GPP–SIF relationship	All

It is not clear which parameter, or set of parameters, is predominantly responsible for the widespread reduction in GPP across many regions and PFTs. Rather, posterior parameter values vary across PFTs for all parameters, with no clear pattern of increase or decrease (Supplementary Table S1; Fig. 4). This is likely due to parameter error correlation (Supplementary Fig. S1) and model equifinality, which arises when multiple sets of parameter values result in a similar fit to the data within the given uncertainties. The *T*_*opt*_ and *T*_*min*_ posterior errors are negatively correlated for all tropical and temperate forest PFTs and C3 plants, and *V*_*cmax*_ is correlated with at least one other parameter for all PFTs except BoND (Supplementary Fig. S1). *T*_*opt*_ has increased for all PFTs that showed the strongest reduction in GPP, and *V*_*cmax*_ has decreased for all boreal PFTs that contribute to a large reduction in GPP across high northern latitudes (Fig. 4). The increase in *V*_*cmax*_ for C4 grasses could be responsible for the increase in GPP in semi-arid regions, together with the reduction in *K*_*pheno,crit*_ that causes an earlier start to the growing season. It is likely that a combination of all photosynthesis-related parameters, in addition to parameters that control the amount of leaf biomass available for C assimilation (*SLA*, *LAI*_*max*_ and *K*_*LAI,happy*_), are responsible for the reduction in GPP magnitude for most PFTs. The notable increase in *K*_*pheno,crit*_ (later start to the growing season) for BoBD trees may also explain the high reduction in mean annual GPP for this PFT (see Supplementary Table S2).

The optimisation did not result in a high variability across PFTs in the posterior value of the slope (*SIF*_*a*_) parameter between SIF and GPP (Fig. 4). The mean posterior *SIF*_*a*_ value across all PFTs (~0.21) is consistent with the mean slope across biomes (~0.17) derived by Guanter L. *et al*.^17^, even though there were differences in the SIF data (GOME-2 vs GOSAT), retrieval algorithm (statistical approach vs in-filling of solar Fraunhofer lines), and GPP model (TBM vs MTE upscaled fluxnet product) used in each analysis. The standard deviation in posterior *SIF*_*a*_ values derived in this study is approximately double that calculated in Guanter L. *et al*.^17^. *SIF*_*a*_ and *SIF*_*b*_ were not strongly correlated with each other for all PFTs, although the slope parameter was correlated with at least one other parameter for all PFTs (Supplementary Fig. S1). The highest correlations (≥0.5) between *SIF*_*a*_ and *SIF*_*b*_ were found for tropical and temperate broadleaved trees and C4 plants (Supplementary Fig. S1). Strong correlations between *SIF*_*a*_ and *SIF*_*b*_ may either result from their relatively uninformative prior bounds (see Methods), compared to values presented in other studies (e.g. ref.^17^), or from high uncertainty in the SIF data. For PFTs that had the highest reduction in GPP (TeBE and boreal PFTs) the slope parameter was typically correlated with one other photosynthesis-related parameter (*V*_*cmax*_ and *F*_*stress,h*_). However, it can be difficult to interpret or place too much confidence in conclusions drawn from the error covariance matrix (Supplementary Fig. S1) for parameters in complex, process-based TBMs, given all the cross-correlations between parameters. The SIF-GPP slope and intercept parameters also account for all fluorescence-related processes not represented in the model, as well as possible temporal and spatial scale mismatches between the model and data. A small number of parameters were ‘edge-hitting’ (5/172 in total), which suggests that missing processes in the model could partially account for remaining model-data misfit.

## Discussion

Within the confines of the current data assimilation set-up and mechanistic representation of the physical and biochemical processes, the SIF data strongly constrained and reduced the simulated global mean annual GPP in the ORCHIDEE model. This reduction in global GPP partially accounts for the presumed positive bias in model GPP compared to data-driven estimates such as JUNG^18^. It is impossible to fully validate the posterior global mean annual GPP estimate, or its trend over time, given the ongoing debate in the literature as to the most realistic value (see Anav A. *et al*.^4^ for further information); however, our results are in line with previous studies, as previously discussed. We do not aim to provide a definitive estimate of the most likely regional to global GPP values; rather, our objective was to determine whether SIF data could provide information for constraining the parameters and processes in model-based estimates of GPP. This, in turn, can provide a more rigorous, statistical and integrated model-data quantification of global mean annual GPP. Here, the SIF data were able to provide a strong constraint on the GPP magnitude in the ORCHIDEE TBM. We suggest this is a potentially important result, given the high degree of spread in GPP magnitude across TBMs is one of the principle sources of uncertainty in global GPP estimates^4^. Furthermore, our results suggest that optimising model GPP using SIF data can modify the finer-scale spatial gradients across tropical and pan-Eurasian biomes that studies have shown to be considerably different among TBMs^4^. Finally, this study has shown that SIF data can adjust the phenology-related ORCHIDEE TBM parameters in addition to those related to C assimilation. This should aid in correcting known biases in TBMs due to incorrect growing season length^3^. However, we suggest that SIF data could be used in combination with satellite-derived vegetation indices (VI, e.g. NDVI) for biomes in which the dynamics of C assimilation and leaf dynamics may be decoupled; for example, this is the case for evergreen forests^35^ and semi-arid ecosystems that controlled by moisture limitation^36^. Furthermore, it is likely that higher temporal resolution (ideally daily) VI and SIF data are needed to better approximate the short timescales associated to leaf onset and senescence.

We do not attempt explore all options in the data assimilation in order to provide the best optimisation set-up. However, the current assimilation set-up is largely similar to previous studies using FLUXNET net CO_2_ fluxes^11^ and NDVI^14^ to constrain the C fluxes and leaf phenology in the ORCHIDEE model, respectively. These studies used the same DA system, the same version of ORCHIDEE, and a similar number of sites. The only differences from previous assimilations were the number of parameters included in the optimisation (given the different processes being optimised) and the site locations (given the different datasets). The parameter prior values, uncertainties, and parameter bounds were the same. A comparison of the global GPP resulting from these assimilations shows that the SIF optimisation appears to result in larger decrease in global mean annual GPP than either of the aforementioned datasets (compare Supplementary Fig. S2d with Fig. S2e and f) due to a greater reduction in overall GPP magnitude worldwide and a decrease in peak growing season GPP in the northern extratropics (Supplementary Fig. S3). Furthermore, in contrast to the SIF optimisation, FLUXNET and NDVI data did not result in a distinct change in latitudinal GPP gradients (Supplementary Fig. S4). These results suggest that SIF data will be extremely useful in constraining GPP at global scale via parameter optimisation and state estimation. A full factorial experiment testing C cycle–related observations, including SIF, FLUXNET net and gross C flux data, and satellite-derived indices of vegetation dynamics (e.g. NDVI) would be needed to fully determine whether SIF data can provide a greater constraint on GPP magnitude and seasonality than other sources of data; however, this was beyond the scope of our study. We expect that SIF will provide a greater constraint on GPP than FLUXNET and satellite data alone.

Our work also demonstrates that it is not necessary to have an explicit mechanistic photosynthesis–fluorescence model in order to exploit SIF data to constrain estimates of GPP. The slope (*SIF*_*a*_) and intercept (*SIF*_*b*_) parameters of the simple linear SIF-GPP relationship are able to account for such missing processes, as well as any biases in the SIF data magnitude, or a mismatch in temporal and spatial scale between the model and data. However, due to correlations between *SIF*_*a*_ or *SIF*_*b*_ and the other model parameters, we have to exercise caution when using the derived photosynthesis and phenology parameter values in future simulations under conditions of changing climate. Implementing a mechanistic representation of fluorescence at leaf scale, and the scaling to canopy, may result in more realistic parameter values for use in future simulations; however this would increase the number of parameters that need to be optimised. The ability of the optimisation algorithm to find unique (un-correlated) values for a greater number of parameters would likely require a higher observation information content; this may or not be achievable with the currently available satellite-derived global SIF products.

Note that it is not a requirement that a simple empirical model between SIF and GPP takes a linear form, as used in this study. It is possible to implement more complex empirical SIF-GPP models in TBMs that are specific to each biome or dependent upon climate. Although recent studies have added weight to the notion that a linear relationship between GPP and SIF exists at multiple scales^15,22,23,25,27^, much work still needs to be done to confirm the consistency of the linear relationship under certain circumstances. These include periods of drought stress, complex canopy structures, and under different viewing and illumination geometries, among other factors. However, due to its close association with photosynthesis, we expect that SIF may provide even more information for constraining GPP than satellite-derived measures of vegetation dynamics (e.g. NDVI, EVI) in periods of water stress^36^. Therefore, future data assimilation experiments may take advantage of such aspects of SIF data to examine and constrain model behaviour in response to drought.

Finally, while the SIF data provided sufficient information to constrain parameters across the majority of PFTs – resulting in strong reductions in GPP in the northern latitudes and tropical regions – structural deficiencies or missing processes in the model may prevent any data assimilation experiment from being able to fully ‘correct’ modelled GPP. Examples in this version of ORCHIDEE include the lack of explicit phenology for evergreen trees, or nutrient (N and P) limitation on photosynthesis. Furthermore, given that parameters are typically PFT-dependent in most TBMs, spatial patterns of GPP require that the underlying PFT map is accurate. However, algorithms for deriving global maps of vegetation distribution are inherently uncertain; this in turn can result in considerable spread in model GPP estimates^37^. Issues related to prescribed PFT fractions may become obsolete if future model versions move from a discrete PFT based to a plant functional traits-based approach to simulating vegetation distributions (e.g. Bodegom P. M. van *et al*.^38^). As with any parameter data assimilation experiment, the full potential of SIF data for constraining GPP in global-scale TBMs may only be realised once forcing and model structural deficiencies are identified and resolved.

## Methods

### ORCHIDEE terrestrial biosphere model

**Figure 5:**
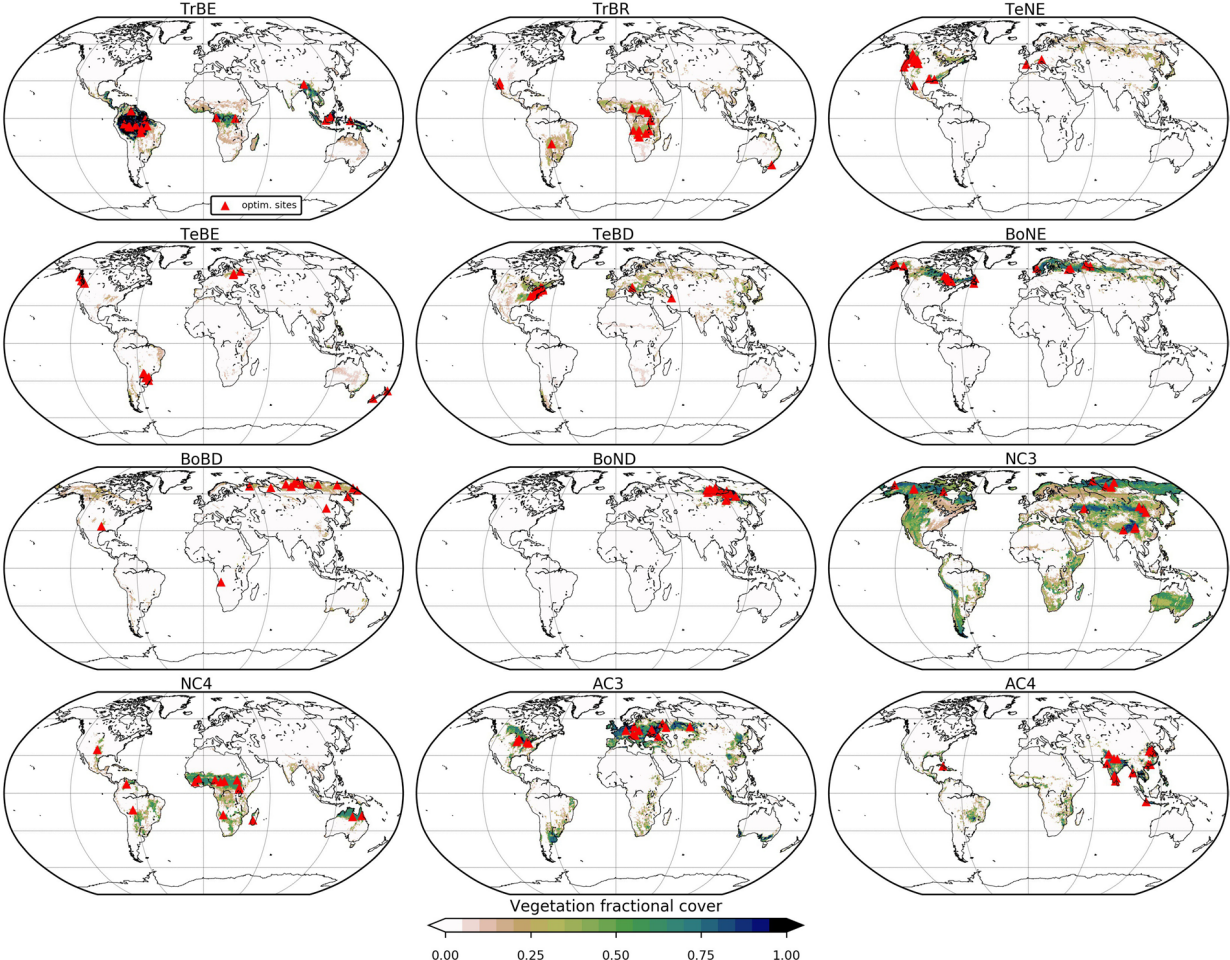
Global spatial distributions of vegetation fractional cover for the 12 ORCHIDEE PFTs optimised in this study (TrBE: tropical broadleaved evergreen; TrBR: tropical broadleaved raingreen; TeNE: temperate needleleaved evergreen; TeBE: temperate broadleaved evergreen; TeBD: temperate broadleaved deciduous; BoNE: boreal needleleaved evergreen; BoBD: boreal broadleaved deciduous; BoND: boreal needleleaved deciduous; NC3: natural C3 grass; NC4: natural C4 grass; AC3: C3 crops (agriculture); AC4: C4 crops (agriculture)). Red triangles mark the location of the optimisation sites. The maps were created from the ORCHIDEE model simulations performed in this study using the Python programming language v2.7.13 (Python Software Foundation – available at http://www.python.org) Matplotlib (v2.0.2) plotting library^54^ with the Basemap Toolkit (http://matplotlib.org/basemap/). See Section on Data Availability for the ORCHIDEE model licence information and ORCHIDEE code availability.

ORCHIDEE is a global process-based terrestrial biosphere model (TBM) that calculates carbon, C, water and energy fluxes between the land surface and the atmosphere at a half-hourly time step^39^. It is the land surface component of the IPSL Earth System Model^40^. In this study, we use the ‘AR5’ version that contributed to the IPCC Fifth Assessment Report^41^. In the biogeochemical component of the model, C is assimilated via photosynthesis depending on light availability, CO_2_ concentration and soil moisture. Photosynthesis is modelled using functions based on Farquhar G. D. *et al*.^42^ for C3 plants and Collatz G. *et al*.^43^ for C4 plants. A prognostic leaf area index (LAI) is calculated on a daily time step. The phenology models control the timing of leaf onset and senescence and have been described in detail in MacBean N. *et al*.^14^. Note that there is no specific phenology model associated to evergreen ecosystems – leaf turnover is simply a function of climate and leaf-age. The plant functional type (PFT) concept in TBMs groups plants according to their physiological behaviour under similar climatic conditions. In ORCHIDEE each grid cell is described by the fractional coverage of all twelve vegetation-related PFTs plus bare soil (see Fig. 5 for PFT names and acronyms).

### Datasets

#### Solar-induced fluorescence (SIF)

In this study, we used near-infrared SIF derived from data acquired by the Global Ozone Monitoring Experiment-2 (GOME-2) instrument on board EUMETSAT’s polar orbiting Meteorological Operational Satellite-A (MetOp-A). The retrieval, described by Köhler P. *et al*.^44^, essentially disentangles the SIF emission from various spectral features related to atmospheric absorption, scattering, and surface reflectance. In particular, we use the monthly aggregated SIF data between 2007–2011 with a spatial resolution of 0.5° × 0.5° in our analysis.

#### Gross primary productivity (GPP)

Global gridded GPP products described in Jung M. *et al*.^18^ were used as an independent benchmark of the prior and posterior simulated GPP. The global gridded products are derived using a data-driven statistical Model Tree Ensemble (MTE) approach to upscale *in situ* eddy covariance C flux measurements from FLUXNET sites (http://fluxnet.fluxdata.org/)^45^. We used the May 2012 version of the gridded GPP product, hereafter referred to as ‘JUNG’. Gross CO_2_ fluxes (GPP and total ecosystem respiration) were derived from net CO_2_ fluxes using a flux partitioning method^46^. Although this product is data-driven, it relies heavily on machine learning and empirical models that contain their own assumptions. We do not therefore use this product to evaluate the model simulations, but rather for a comparison with another global scale product.

### Data assimilation framework and methodology

#### SIF-GPP linear relationship

In this version of the model, we assumed that SIF scales linearly with GPP, via the equation:

1 $$SIF=aGPP+b$$

We assumed this relationship holds at daily to monthly time steps and at the 0.5° spatial resolution of the simulations carried out in this study. As described in the introduction, this assumption is based on the results of several studies, which found that while instantaneous measurements of SIF and GPP at the leaf level are non-linear and dependent on vegetation type, the relationship becomes linear upon aggregation to the canopy and at daily to seasonal time-scales^15,22,23,25,27^. In the model, SIF is calculated at a daily time step, and a and b are PFT-specific parameters (*SIF*_*a*_ and *SIF*_*b*_ in Table 2) were optimised in the assimilation system. We assume that *SIF*_*a*_ and *SIF*_*b*_ absorb biases in the SIF retrieval algorithm, mismatches in magnitude between the model and data due to temporal and spatial averaging, and ORCHIDEE model structural issues (for example lack a fluorescence module and clear sky vs diffuse radiation effects).

#### Data assimilation system description

The ORCHIDEE Data Assimilation (DA) System (http://orchidas.lsce.ipsl.fr) is based on a variational DA system that has been described in detail in previous studies^8,13,14^. It follows a Bayesian framework, in which the optimal parameter vector *x* can be found by minimizing the following cost-function *J(x)*, assuming that the probability distribution functions (PDFs) of the model parameter and observation uncertainties are Gaussian^47^:

2 $${J}({\boldsymbol{x}})=\frac{1}{2}[{({H}({\boldsymbol{x}})-{\boldsymbol{y}})}^{{\boldsymbol{T}}}{{\bf{R}}}^{-1}({\boldsymbol{H}}({\boldsymbol{x}})-{\boldsymbol{y}})+{({\boldsymbol{x}}-{{\boldsymbol{x}}}_{{\boldsymbol{b}}})}^{{\boldsymbol{T}}}{{\bf{P}}}_{{\boldsymbol{b}}}^{-1}({\boldsymbol{x}}-{{\boldsymbol{x}}}_{{\boldsymbol{b}}})]$$

where *x*_*b*_ are the *a priori* parameter values, **P**_*b*_ the *a priori* uncertainty matrix of the parameters, *y* is the observation vector, *H(x)* the model outputs, given parameter vector *x*, and **R** the uncertainty matrix of the observations (including observation and model errors). In this case, *x* is a vector of the parameters listed in Table 2), and *H(x)* is the simulated SIF derived from equation (1). The observation and model errors are assumed to be uncorrelated in space and time, and parameters are assumed to be independent; hence **R** and **P**_*b*_ are diagonal matrices. The cost function is iteratively minimised using the gradient-based L-BFGS-B algorithm^48^, which allows for fixed parameter bounds. In this version of the model the gradient of the *J(x)* (termed the Jacobian) is estimated using a finite difference method. To improve the efficiency of the L-BFGS-B algorithm, the range of variation of the parameters is standardised by scaling parameter vector, *x*, their priors and uncertainties following: ${\boldsymbol{x}}^{\prime} ={{\bf{P}}}_{b}^{-\frac{1}{2}}({\boldsymbol{x}}-{{\boldsymbol{x}}}_{b})$^49^.

In order to quantify the constraint brought by the assimilation on the model parameters and state variables, we calculate the posterior parameter error covariance matrix, **P**_*post*_ using the Jacobian of the model at the minimum of *J(x)*, ${{\bf{H}}}_{\infty }$, following Tarantola A.^47^ :

3 $${{\bf{P}}}_{{\rm{post}}}={[{{\bf{H}}}_{\infty }^{T}{{\bf{R}}}^{-1}{{\bf{H}}}_{\infty }+{{\bf{P}}}_{b}^{-1}]}^{-1}$$

**P**_*post*_ is then propagated onto the model state variables (e.g. GPP) space given the following matrix product and the hypothesis of local linearity^47^:

4 $${{\bf{R}}}_{{post}}={\bf{H}}{{\bf{P}}}_{{post}}{{\bf{H}}}^{T}$$

The square root of the diagonal elements of **R**_*post*_ corresponds to the posterior error (standard deviation, 1*σ*). In order to assess the improvement brought by the assimilation, the error reduction is determined as 1 − (**R**_*post*_ / **R**_*prior*_).

#### Optimised parameters and derivation of prior values and uncertainties

We optimised model parameters involved in the calculation of seasonal uptake of carbon (GPP) in ORCHIDEE, including parameters related to photosynthesis and leaf phenology, as well as *SIF*_*a*_ and *SIF*_*b*_ parameters in equation (1) (Table 2). All parameters were optimised for each PFT. For the photosynthesis and phenology parameters, the prior values were taken from the ORCHIDEE standard version (Supplementary Table S2). The parameter maximum and minimum bounds were based on literature and elicitation of ‘expert’ knowledge. To derive the *SIF*_*a*_ and *SIF*_*b*_ prior values and bounds we first examined the distribution of the slope and intercept parameters resulting from a linear regression between the SIF data and both the prior ORCHIDEE simulation and the ‘JUNG’ product for each PFT. Considering these distributions, the slope parameter bounds were set to −0.5 to 1, and the intercept parameter bounds were set to −1.5 to 2.5. The prior values for both *SIF*_*a*_ and *SIF*_*b*_ were set to 0.25. The prior uncertainty for all parameters was set at 40% of the parameter range following Kuppel S. *et al*.^50^.

#### PFTs optimised and site selection

We performed a multi-site optimisation, in which all sites are included in the same optimisation, following^11,14,50^, for each of the 12 vegetated PFTs. Following MacBean N. *et al*.^14^ we selected 15 grid cells that fulfilled several constraints to optimise for each PFT (Fig. 5). This was achieved by a random sampling of grid cells with a fractional coverage above the given threshold. First, the grid cells had to contain a high fraction of the PFT in question. This was mostly >0.6 except for the BoBD PFT where the fractional cover is never >0.3. Second, the site locations had to be representative of the overall spatial distribution of each PFT. No more than 15 grid cells per PFT were chosen in this study for two reasons: (1) the lack of representative grid cells for certain PFTs at this spatial scale; and (2) the computational time required to perform a multi-site assimilation.

### Model setup and experiments performed

#### Model setup

In this study, ORCHIDEE is used in forced offline mode and driven by 6-hourly CRU-NCEP v5 meteorological fields at 0.5 × 0.5° resolution corresponding to the resolution of the SIF data. We used the Olson land cover classification to prescribe PFT fractions^51^ and the soil map from Zobler L.^52^ to prescribe texture classes for each grid cell. The impacts of land use change, forest management, harvesting and fires were not included.

#### Assimilation experiments and posterior model simulation

For all selected sites, we performed a ‘spin-up’ of the vegetation state to ensure the model reaches an equilibrium for GPP. The spin-up simulation was used forcing data cycled over the 50 years prior to the observation period (1956–2006). Next, we performed the assimilation simulation for the observation period (2007–2011). Finally, global-scale simulations were performed with the prior and posterior parameter vectors in order to evaluate the impact of the optimisation on the regional and global simulated GPP.

#### Biome level posterior analyses

Biome level analyses were based on the Köppen Geiger (KG) climate classification scheme derived by Peel M. C. *et al*.^53^. We grouped the main climate classes in the following way: ‘tropical biomes’ correspond to class A in KG scheme; ‘arid biomes’ correspond to class B; and ‘temperate and boreal biomes’ encompass classes C to E.

### Data availability

The GOME-2 level 1B product, containing necessary radiance spectra and daily solar irradiance measurements, was obtained from EUMETSAT’s Data Centre (https://www.eumetsat.int/website/home/Data/DataDelivery/EUMETSATDataCentre/index.html). The SIF retrieval method using the GOME-2 level 1B product is described in Köhler *et al*. (2015). The 6-hourly CRU-NCEP v5 meteorological fields at 0.5 × 0.5° resolution are available from https://esgf.extra.cea.fr/thredds/catalog/store/p529viov/cruncep/V5_1901_2013/catalog.html. The GPP MTE data (referred to as the ‘JUNG’ product in this study) were downloaded from the GEOCARBON data portal (FileID 66) in compliance with the Data Usage Agreement (https://www.bgc-jena.mpg.de/geodb/projects/Home.php).

The ORCHIDEE model is under a free software license (CeCILL; see http://www.cecill.info/index.en.html). The ORCHIDEE model source code is visible here: https://forge.ipsl.jussieu.fr/orchidee/browser/tags/ORCHIDEE_1_9_6.

## Electronic supplementary material

Supplementary Information
