# Author Correction: Strong constraint on modelled global carbon uptake using solar-induced chlorophyll fluorescence data

**DOI:** 10.1038/s41598-018-28697-z

**Published:** 2018-07-05

**Authors:** Natasha MacBean, Fabienne Maignan, Cédric Bacour, Philip Lewis, Philippe Peylin, Luis Guanter, Philipp Köhler, Jose Gómez-Dans, Mathias Disney

**Affiliations:** 1 Laboratoire des Sciences du Climat et de l’Environnement, LSCE/IPSL, CEA-CNRS-UVSQ, Université Paris-Saclay, F-91191 Gif-sur-Yvette, France; 2 Present Address: University of Arizona, School of Natural Resources and the Environment, Tucson, Arizona 85719 USA; 3 NOVELTIS, Labège, France; 4 Department of Geography, University College London, Pearson Building, Gower Street, London, WC1E 6BT, London, UK; 5 NERC National Centre for Earth Observation (NCEO), Swindon, UK; 6 Helmholtz Centre Potsdam-GFZ German Research Centre for Geosciences, Potsdam, Germany; 7 California Institute of Technology (Caltech), Division of Geological and Planetary Sciences, Pasadena, CA USA

Correction to: *Scientific Reports* 10.1038/s41598-018-20024-w, published online 31 January 2018

In this Article, reference 23 is incorrectly cited in its third instance. The correct reference appears below as reference 1, and should appear in the Introduction as below:

“Meanwhile, several modelling groups have used SIF data to optimise fluorescence model parameters^23^”

should read:

“Meanwhile, several modelling groups have used SIF data to optimise fluorescence model parameters^1^”
