# Supplementary material for: Strong constraint on modelled global carbon uptake using solar-induced chlorophyll fluorescence data
Source: Sci Rep. 2018 Jan 31;8:1973. doi: 10.1038/s41598-018-20024-w (PMC5792553; doi:10.1038/s41598-018-20024-w)

Supplementary Information for

Strong constraint on modelled global carbon uptake using solar-induced chlorophyll fluorescence data

Natasha MacBean[1,2*], Fabienne Maignan[1], Cédric Bacour[3], Philip Lewis[4,5,]

Philippe Peylin[1], Luis Guanter[6], Philipp Köhler[7], Jose Gomez-Dans[4,5],

Mathias Disney[4,5]

[1]Laboratoire des Sciences du Climat et de l'Environnement, LSCE/IPSL, CEA-CNRS-UVSQ, Université Paris-Saclay, F-91191 Gif-sur-Yvette, France

[2]Now at University of Arizona, School of Natural Resources and the Environment, Tucson, Arizona, 85719, USA.

[3]NOVELTIS, Labège, France

[4]Department of Geography, University College London, Pearson Building, Gower Street, London WC1E 6BT, London, United Kingdom

[5]NERC National Centre for Earth Observation (NCEO)

[6]Helmholtz Centre Potsdam - GFZ German Research Centre for Geosciences, Potsdam, Germany

[7]California Institute of Technology (Caltech), Division of Geological and Planetary Sciences, Pasadena, CA, USA.

.

*_Corresponding author address_: Natasha MacBean, University of Arizona, School of Natural Resources and the Environment, Tucson, Arizona, 85719, USA.
E-mail: nlmacbean@gmail.com

Supplementary Table S1: Prior and posterior (bold font) parameter values for each parameter and each PFT. A dash denotes that a parameter is not dependent on that PFT, and therefore not optimized. (See Fig. 5 for PFT acronym descriptions).

**Parameter**	**PFT**											
	TrBE	TrBR	TeNE	TeBE	TeBD	BoNE	BoBD	BoND	NC3	NC4	AC3	AC4
V_{cmax}	65<br>**66.54**	65<br>**69.99**	35<br>**46.35**	45<br>**46.84**	55<br>**54.02**	35<br>**26.29**	45<br>**38.51**	35<br>**28.71**	70<br>**39.86**	70<br>**81.53**	70<br>**61.44**	70<br>**67.18**
$G_{s,slope}$	9<br>**10.37**	9<br>**10.26**	9<br>**9.44**	9<br>**11.27**	9<br>**9.16**	9<br>**6.3**	9<br>**10.13**	9<br>**11.59**	9<br>**6.6**	3<br>**2.34**	9<br>**6.67**	3<br>**3.33**
T_{opt}	37<br>**42.82**	37<br>**32.15**	25<br>**30.42**	32<br>**37.55**	26<br>**26.35**	25<br>**30.2**	25<br>**33**	25<br>**32.01**	27.25<br>**34.33**	36<br>**38.87**	30<br>**29.05**	36<br>**35.61**
T_{min}	2<br>**6.92**	2<br>**6.83**	-4<br>**-0.81**	-3<br>**1.83**	-2<br>**-1.75**	-4<br>**-6.59**	-4<br>**-5.87**	-4<br>**-3.09**	-3.25<br>**0.26**	13<br>**16.24**	-5<br>**-4.2**	13<br>**11.53**
T_{max}	55<br>**59.26**	55<br>**54.4**	38<br>**39.65**	48<br>**44.21**	38<br>**37.74**	38<br>**34.87**	38<br>**36.03**	38<br>**35.74**	41.12<br>**36.63**	55<br>**52.16**	45<br>**45.58**	55<br>**53.64**
$F_{stress,h}$	6<br>**6.85**	6<br>**7.21**	6<br>**4.39**	6<br>**6.47**	6<br>**5.8**	6<br>**5.61**	6<br>**7.33**	6<br>**2.13**	6<br>**4.69**	6<br>**6.81**	6<br>**3.16**	6<br>**7.81**
SLA	0.015<br>**0.019**	0.026<br>**0.014**	0.009<br>**0.007**	0.02<br>**0.017**	0.026<br>**0.028**	0.009<br>**0.012**	0.026<br>**0.013**	0.019<br>**0.026**	0.026<br>**0.035**	0.026<br>**0.019**	0.026<br>**0.021**	0.026<br>**0.036**
LAI_{max}	7<br>**6.82**	7<br>**5.85**	5<br>**3.84**	5<br>**5.69**	5<br>**4.89**	4.5<br>**5.02**	4.5<br>**5.54**	3<br>**2.85**	2.5<br>**3.16**	2.5<br>**2.64**	5<br>**7**	5<br>**5.54**
$K_{LAI,happy}$	0.5<br>**0.44**	0.5<br>**0.57**	0.5<br>**0.46**	0.5<br>**0.49**	0.5<br>**0.51**	0.5<br>**0.66**	0.5<br>**0.53**	0.5<br>**0.45**	0.5<br>**0.38**	0.5<br>**0.46**	0.5<br>**0.41**	0.5<br>**0.61**
$K_{pheno,crit}$	-	-	-	-	1.0<br>**0.98**	-	1.0<br>**1.71**	1.0<br>**1.18**	1.0<br>**0.86**	1.0<br>**0.75**	1.0<br>**1.74**	1.0<br>**1.23**
M_{Tmin}	-	50<br>**15.44**	-	-	-	-	-	-	35<br>**28.37**	35<br>**50.71**	75<br>**64.71**	75<br>**99.81**
$L_{age,crit}$	730<br>**828**	180<br>**194**	910<br>**1086**	730<br>**742**	180<br>**175**	910<br>**765**	180<br>**213**	180<br>**90**	120<br>**98**	120<br>**124**	90<br>**127**	90<br>**128**
T_{senes}	-	-	-	-	12<br>**12.54**	-	7<br>**3.53**	2<br>**12**	-1.38<br>**-0.11**	5<br>**5.62**	5<br>**9.96**	10<br>**13.08**

$M_{senes,nosenes}$	-	-	-	-	-	-	-	-	0.3<br>**0.33**	0.3<br>**0.55**	0.3<br>**0.54**	0.3<br>**0.31**
L_{fall}	-	10<br>**47**	-	-	10<br>**12**	-	10<br>**31**	10<br>**22**	-	-	-	-
SIF_a	0.25<br>**0.14**	0.25<br>**0.09**	0.25<br>**0.25**	0.25<br>**0.35**	0.25<br>**0.22**	0.25<br>**0.21**	0.25<br>**0.31**	0.25<br>**0.32**	0.25<br>**0.31**	0.25<br>**0.06**	0.25<br>**0.17**	0.25<br>**0.03**
SIF_b	0.25<br>**1.13**	0.25<br>**1.26**	0.25<br>**0.19**	0.25<br>**-0.09**	0.25<br>**0.17**	0.25<br>**-0.07**	0.25<br>**-0.58**	0.25<br>**-0.01**	0.25<br>**0.12**	0.25<br>**0.8**	0.25<br>**0.24**	0.25<br>**1.21**

Supplementary Table S2: Prior and posterior mean annual GPP in $kgCm^{-2}yr^{-1}$ at global scale, across biomes and per PFT. Biomes are based on the Köppen-Geiger (KG) classification based on Peel M. C. *et al.*[54]. PFT means are calculated across all grid cells with a vegetation fraction greater than described in Section 3.3.4. (See Fig. 5 for PFT acronym descriptions).

Latitudinal region / PFT	Prior mean GPP ($kgCm^{-2}yr^{-1}$)	Posterior mean GPP ($kgCm^{-2}yr^{-1}$)
Global	1.31	1.07
Temperate + boreal KG biome	1.16	0.82
Boreal KG Biome	0.9	0.56
Temperate KG Biome	2.07	1.72
Tropical KG biome	3.32	3.09
Arid KG biome	0.3	0.27
TrBE	3.9	3.36
TrBR	2.26	1.99
TeNE	1.78	1.36
TeBE	2.05	1.22
TeBD	2.24	2.08
BoNE	1.28	0.75
BoBD	1.88	0.87
BoND	1.06	0.23
NC3	0.7	0.24
NC4	2.21	2.37
AC3	2.45	2.19
AC4	3.45	3.63

Supplementary Table S3: Prior and posterior mean monthly correlation between GPP and SIF (2007-2011) averaged across all grid cells with a vegetation fraction greater than described in Section 3.3.4. (See Fig. 5 for PFT acronym descriptions).

PFT	Prior mean monthly GPP-SIF correlation	Posterior mean monthly GPP-SIF correlation
TrBE	0.05	0.06
TrBR	0.8	0.84
TeNE	0.92	0.95
TeBE	0.78	0.81
TeBD	0.98	0.98
BoNE	0.95	0.95
BoBD	0.77	0.91
BoND	0.96	0.94
NC3	0.84	0.9
NC4	0.83	0.82
AC3	0.66	0.78
AC4	0.63	0.62

Supplementary Figure S1: Posterior parameter covariance matrices for all PFTs (see Fig. 5 for PFT acronym descriptions). Covariance matrices were calculated for the assimilation window (2007-2011) across all 15 site simulations for each PFT.

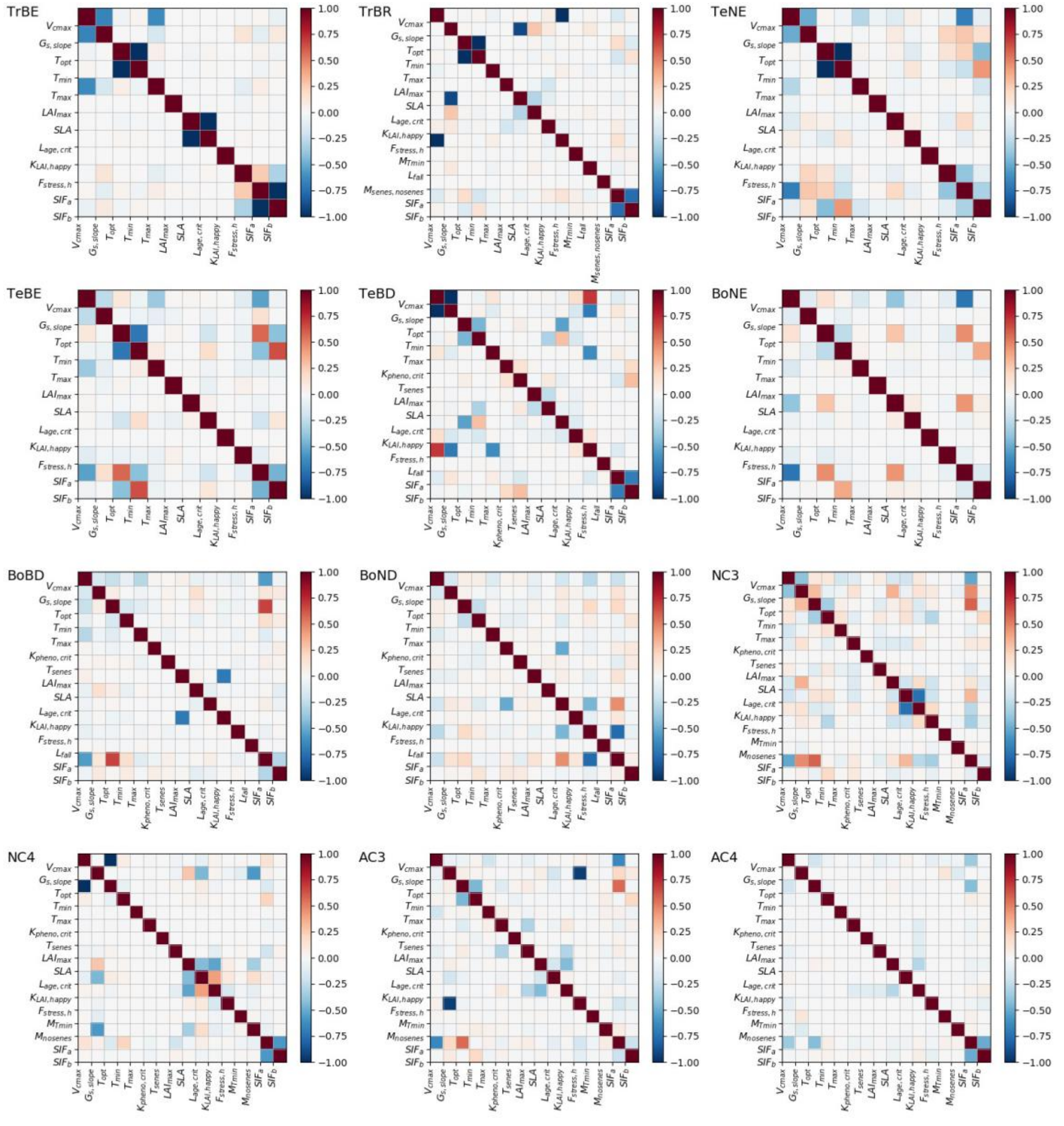

Supplementary Figure S2: Global mean annual sum (2007-2011) and spatial distribution of: a) GOME-2 SIF; b) JUNG up-scaled FLUXNET data-driven GPP product [Jung et al. 2011]; c) ORCHIDEE prior GPP; d) ORCHIDEE posterior GPP; e) ORCHIDEE posterior GPP resulting from the optimization with NDVI data[14]; f) ORCHIDEE posterior GPP resulting from the optimization with FLUXNET data[11]. The maps were created from the ORCHIDEE model simulations performed in this study and in refs 11 and 14, and the JUNG dataset, using the Python programming language v2.7.13 (Python Software Foundation – available at http://www.python.org) Matplotlib (v2.0.2) plotting library[55] with the Basemap Toolkit (http://matplotlib.org/basemap/). See Section on Data Availability for JUNG product availability, the ORCHIDEE model licence information and ORCHIDEE code availability.

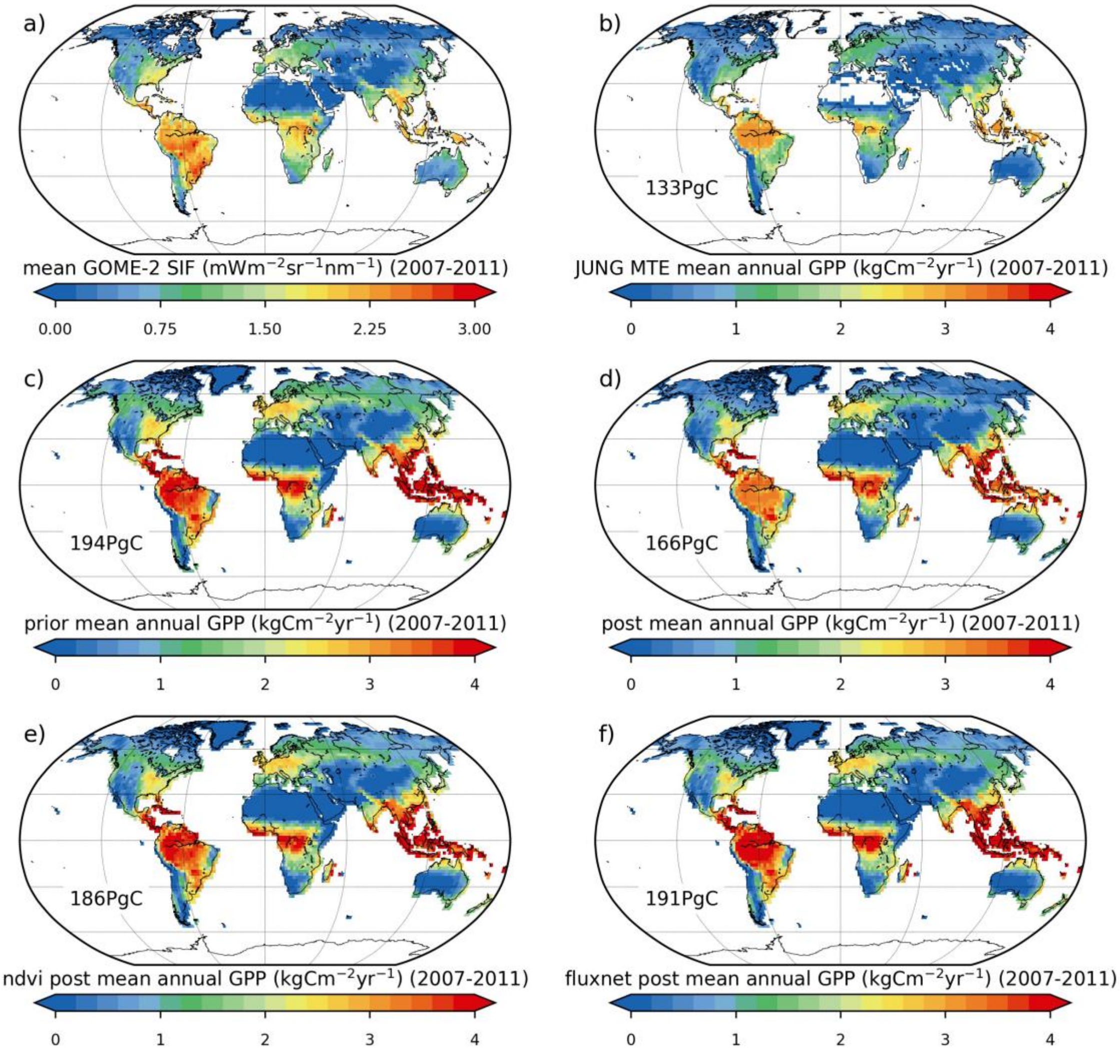

Supplementary Figure S3: Mean monthly GPP seasonal cycle over 2007-2011 period (PgC/month) for: a) temperate and boreal Köppen-Geiger (KG) biomes (approximately equivalent to northern hemisphere >60°N); b) tropical KG biomes (approximately equivalent to tropical latitudes 30°S to 30°N); c) arid KG biomes. The prior simulation is shown in the red curve, and the posterior in blue. The grey curve shows a comparison with the JUNG up-scaled FLUXNET data-driven GPP product by *Jung et al*. [2011]. The dashed yellow and green curves show previous ORCHIDEE posterior simulations from MacBean N. *et al*.[14] and Kuppel S. *et al*.[11], using MODIS NDVI and site-based eddy covariance carbon and water flux data, respectively. Köppen-Geiger classification based on Peel M. C. *et al*.[54].

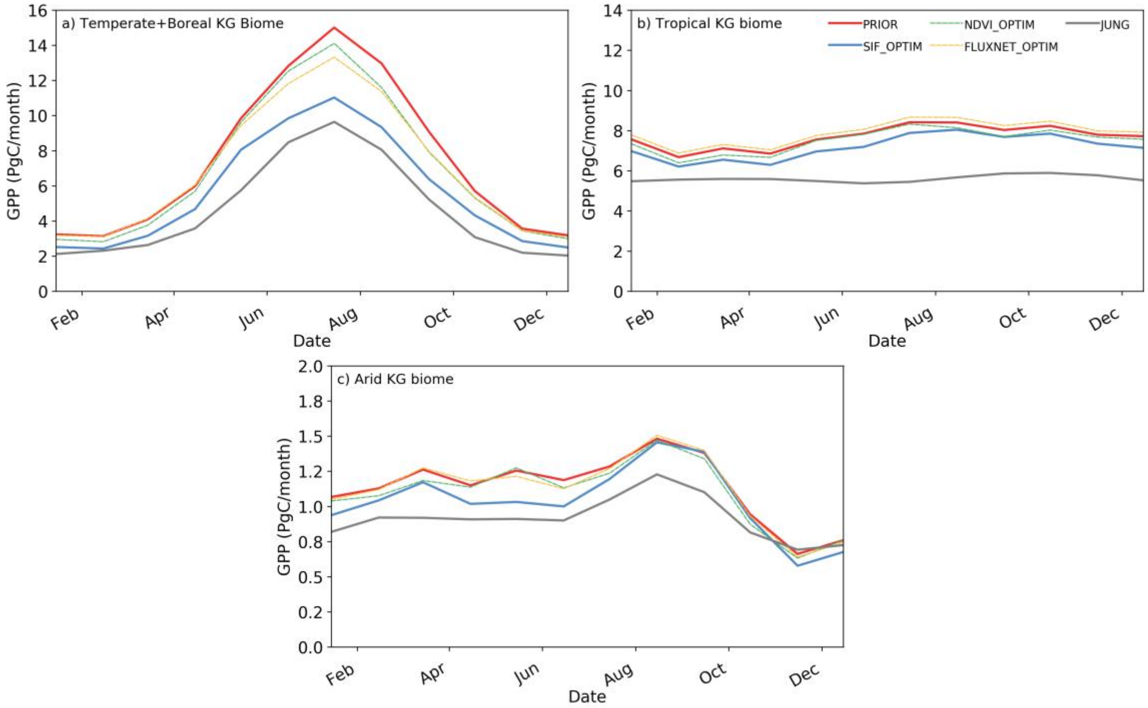

Supplementary Figure S4: Latitudinal plot of mean annual GPP ($kgCm^{-2}yr^{-1}$) over the 2007-2011 period. Red curve: prior simulation; blue curve: posterior resulting from the optimization with SIF data (this study); yellow dashed curve: posterior resulting from the optimization with NDVI data[14]; green dashed curve: the posterior from the optimization with FLUXNET data[11]; and grey: the JUNG product.

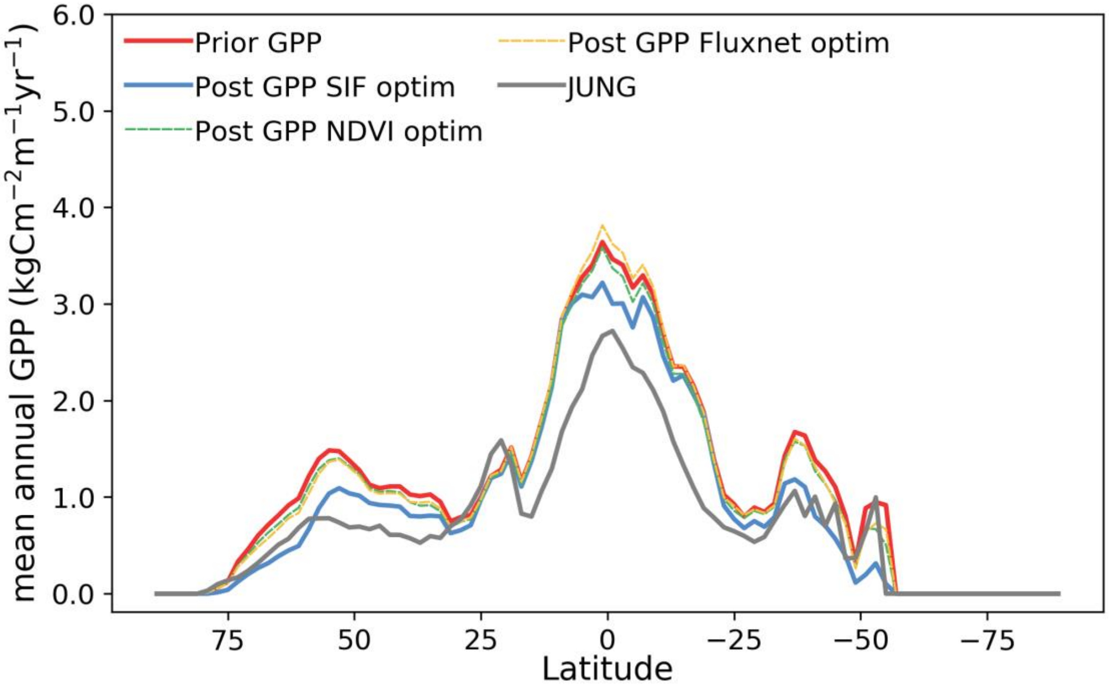

Supplement: Supplementary file 1 — Supplementary Information [file 41598_2018_20024_MOESM1_ESM.pdf]
